# The accuracy and inter-observer reliability of acetate templating in total hip arthroplasty

**DOI:** 10.1186/1753-6561-6-S4-O33

**Published:** 2012-07-09

**Authors:** R Kearney, J O'Byrne, A Shaikh

**Affiliations:** 1 Royal College of Surgeons in Ireland

## Introduction

A number of orthopedic surgeons use traditional acetate x-rays to pre-operatively template in primary total hip arthroplasty (THA). Templating, when successful can prevent the use of oversized or undersized implants. However, ignoring to template can result in many complications. In Cappagh National Orthopedic Hospital Dublin (CNOH), a number of consultant surgeons do not routinely template; while in some cases only the registrars template routinely. However, inter-observer reliability (IOR) between consultants and registrars is unknown. Our study hopes to answer two key questions: How accurate is pre-operative acetate templating in a National Orthopedic Hospital, and when only trainee registrars template, is the accuracy of templating affected?

## Methods

A prospective cohort study was carried out over four weeks on 38 THA patients in CNOH, based on questionnaire responses obtained from two surgeons from the same operating team (senior consultant surgeon and trainee registrar surgeon). X-rays were templated pre-operatively individually by each surgeon using manufacturer supplied transparent acetate template sheets. Each surgeons measurements were noted on the questionnaires. The operative book was reviewed post-operatively to determine the final size of implant used. Surgeon's template acetabular cup, femoral stem and femoral offset sizes were noted and compared with the final implant sizes for accuracy. Both consultant and registrar's predictions were noted and compared with each other for IOR of templating.

## Results

Templating was found to be accurate to within one size of final implant size for 75% of acetabular cups and 91% of femoral stems. Prediction of exact femoral offset size was accurate 91% of the time. Templating also showed strong IOR within one size of corresponding surgeon's template measurements for cup (83%) and stem (100%), as well as exactly matching 92% of the time for femoral offset.

## Conclusions

We conclude that acetate templating is beneficial for both senior consultant and trainee surgeons at gauging within one size of cup and stem implants as well as predicting exact femoral offset size. We would encourage the widespread use of tempalting as it can reduce complications associated with THA and also save both time and money in the the health care service.

